# Is happier music groovier? The influence of emotional characteristics of musical chord progressions on groove

**DOI:** 10.1007/s00426-023-01869-x

**Published:** 2023-08-24

**Authors:** Satoshi Kawase

**Affiliations:** The Faculty of Psychology, Kobe Gakuin University, 518 Arise, Ikawadani-cho, Nishi-ku, Kobe, Hyogo 651-2180 Japan

## Abstract

Specific rhythmic patterns in music have been reported to induce an urge to move with feelings of pleasure or enjoyment, called “groove.” However, it is unclear how the emotional characteristics of music (e.g., happiness or sadness) affect groove. To address this issue I investigated the effects of the emotional characteristics of music on groove by altering the chord progressions accompanying drum breaks composed by a professional composer while manipulating independent tempo and rhythmic patterns. An online listening experiment was conducted using pieces composed by a professional composer but comprising different types of chord progressions that lead to happiness or sadness. Participants evaluated the nine items on a 7-point scale, including urge to move (i.e., groove), felt emotions, *nori*, and liking. The experiment found that: (1) chord progressions that evoke happiness were more likely to induce groove, (2) emotional characteristics did not interact with tempi and syncopation in terms of groove ratings, and (3) the accompaniment of drum breaks enhanced groove in both happy and sad chord progressions. Musical pieces with chord progressions that induce happiness were more likely to evoke groove, namely the urge to move. This implies that considering the emotional characteristics of musical pieces and rhythms is crucial when creating music for movement during rehabilitation, therapy, or dance.

**Supplementary Information:**

The online version contains supplementary material available at 10.1007/s00426-023-01869-x.

## Introduction

### Groove connects music and the body

Groove is a worldwide phenomenon of listening to music, which is defined as the urge to move induced by specific rhythmic patterns or music (Kawase et al., 2001; Madison, 2006; Senn et al., 2016); it is accompanied by positive feelings, such as pleasure or enjoyment (Etaini et al., 2023; Janata et al., 2012; Senn et al., 2020; Stupacher et al., 2016; Witek et al., 2014). Various regions and different cultures bear synonymous terminology for groove; for example, *nori* is a Japanese musical term that is related to the sensation of vertical and horizontal movement (Etani et al., 2018; Kawase & Eguchi, 2010), and “balanço” in Brazilian or “lüpfig” in Swiss German (Senn et al., 2019). Specifically, *nori* is a common everyday word in Japan, which can be connected with words representing the direction in which the body is moving, such as *tate-nori* and *yoko-nori* (Etani et al., 2018; Kawase & Eguchi, 2010). *Tate* represents the vertical direction, whereas *yoko* represents the horizontal direction. *Tate-nori* is frequently used for songs with a relatively fast tempo, such as jumping around, whereas *yoko-nori* is frequently used for songs with a relatively slow tempo (Etani et al., 2018; Kawase & Eguchi, 2010).

Given the strong correlations between music and dance or body movement worldwide (Stevens, 2012), it is important to explore relationships between music and physicality for a comprehensive understanding of human cultural behavior. Such speculations can serve to elucidate not only the correlations between human auditory perception and motor sensation but also universal human cultural behaviors.

### Impacts of rhythmic features on groove

Most previous studies on groove induction have focused on the rhythmic features of music. In an experiment using drum breaks as stimuli, drum breaks were rated as grooviest at a moderate syncopation level (Witek et al., 2014). Varying drum breaks provided an optimal tempo that induced the highest level of groove among listeners (Etani et al., 2018). Furthermore, beat clarity was related to groove induction in a study that used various types of musical pieces and their acoustic characteristics (Madison et al., 2011). Madison and Sioros (2014) indicated that groove is associated with alternations of rhythmic patterns with the same melody. They showed that groove changed by shifts in the timing of the performance, such as in syncopation while playing monophonic melodies. Recent studies on groove have also found interactions between the complexities of rhythm and harmony (Matthews et al., 2019). In other studies, groove was related to timing deviations between instruments that comprise drum breaks, such as a snare drum and a cymbal (Frühauf et al., 2013; Kilchenmann & Senn, 2015), or timing deviations between drums and bass (Prögler, 1995). These findings suggest that groove is linked to various characteristics of rhythm and musical instruments for rhythmic performances, such as drums and bass. Rhythm has strong connections with groove a priori.

Despite the findings of rhythm on groove, however, the associations between rhythm and multifaceted musical components for groove induction factors other than rhythmic features, such as chord progressions or melody, remain unclear. In particular, the relationship between groove and the musical factors that generate emotional experiences, which play an important role in everyday music listening, has rarely been examined.

### Pleasurable feelings accompanying groove

Pleasurable feelings are another characteristic of groove (Etani et al., 2023; Hosken, 2020; Janata et al., 2012; Stupacher et al., 2016; Witek et al., 2014). Witek et al. (2014) showed that pleasure ratings changed in accordance with changes in the syncopation level of drum breaks. They demonstrated an inverted U-shaped correlation between groove induced by syncopation and feelings of pleasure. Furthermore, temporal changes in drum breaks showed a strong association between groove and pleasurable feelings (Etani et al., 2018). Commercially available songs, such as folk and jazz, also provide a highly positive correlation between groove ratings and enjoyment (Janata et al., 2012). Matthews et al. (2020) showed that subjectively perceived grooves are related to the reward system and motor networks of the brain. Senn et al. (2022) examined the psychological model of groove and suggested that a sense of pleasure positively influences the sense of the urge to move.

In addition to groove studies, Burger et al. (2013) demonstrated relationships between emotions expressed by musical tunes and body movements. In this study, participants moved their bodies to commercially available songs expressing various emotions. Their results suggested a positive correlation between happiness, movement complexity, and rotation range, and a positive correlation between acceleration of the head and hands and arousal. Duman et al. (2022) showed that when they collected songs appropriate for physical exercise, those tempi were concentrated at around 120 beats per minute (BPM). In light of these findings, not only the rhythmic aspect but also harmony and melody seem to be correlated with the groove induced by musical pieces. In summary, movement-related groove can be connected to positive feelings.

### Potential relationships between groove and emotion induced by music

Does a musical piece containing elements that can induce pleasurable feelings cause an urge to move one’s body? As music induces various emotions (Eerola et al., 2012) and groove co-occurs with pleasure (Janata et al., 2012), it is presumed that the emotional characteristics of music can be firmly connected with groove. However, although emotion is an essential aspect of music, to the best of our knowledge, no studies have explored whether harmony or melody that induces emotions, such as happiness or sadness (Juslin et al., 2013), can affect groove. Consequently, it remains unknown how groove is influenced by the associations between rhythm, musical factors other than rhythm, and emotions. As musical emotions are provided by multifaceted factors in music such as melody, sound level, timbre, and mode (e.g., Juslin & Timmers, 2010), several questions arise. How are the emotions evoked by non-rhythmic musical factors related to groove? Which factors have a stronger effect on groove, rhythm, or chord progressions?

To address these questions, the present study aimed to investigate the relationship between groove and emotions induced by chord progressions and the interactions induced by chord progressions and rhythmic characteristics. In particular, the present study focused on chord progressions, which have a significant impact on the felt emotions evoked by musical listening (Eerola et al., 2012). Lahdelma and Eerola (2016) showed that variances in musical chords provide different types of emotions to audience members. Their study suggested that musical chords and chord progressions are vertical dimensions. In other words, the musical chords themselves do not affect the temporal structure of the piece. Therefore, by always sounding one chord in the first beat of the measure, this study controlled the emotional properties of the sequence of musical notes without affecting the overall rhythmic structure. This allows the emotional characteristics produced by chord progressions and the rhythmic influence on groove to be manipulated independently.

Among emotions, the present study highlighted happy and sad emotions induced by musical chord progressions, as groove has been observed to arise with pleasurable emotions. Regarding music chords, the major mode, which is related to happy music (Gagnon & Peretz, 2003) induces higher levels of arousal than minor chords (Labbé et al., 2021). Given that groove is related to the flow state (Janata et al., 2012), which is also related to feelings of happiness (Csikszentmihalyi, 2013), it is reasonable to investigate whether groove is felt by elements that evoke pleasant feelings by altering the music chords that may induce emotions. Furthermore, musical emotion is characterized by a combination of several musical elements, such as tempo or mode (Hunter et al., 2010; Juslin & Timmers, 2010). Juslin and Laukka (2004) summarized the relationships between tempo and mode as follows. Happiness: fast tempo, major mode; sadness: slow tempo, minor mode; anger: fast tempo, minor mode; fear: fast tempo, minor mode; tenderness: slow tempo, major mode. Given these optimal combinations of tempo and mode depending on emotion, whether such an interaction exists for groove must be clarified, because tempo and mode are essential factors in groove and emotion, respectively (Etani et al., 2018; Juslin & Laukka, 2004).

### Research questions

To explore the aforementioned issues, this study examined how the emotional characteristics of music influence groove by alternating such characteristics depending on chord progression changes with rhythm control. In addition, the present study investigated how the emotional characteristics of music are associated with rhythmic characteristics, specifically tempo and syncopation. Two online surveys were conducted to answer the following research questions:

- RQ1. Does the emotion (happy and sad) of music evoked by chord progressions affect groove?
- RQ2. Regarding groove ratings, do the emotional valences of chord progressions and rhythmic characteristics interact with each other?
- RQ3. Do chord progressions accompanying drum breaks affect the evaluation of groove?

In “Experiment 1”, to induce different levels of happy and sad emotions, I combined different chord progressions played by piano with the same rhythmic patterns in the online listening survey. Subsequently, I examined whether the emotional characteristics induced by chord progressions influence groove ratings.

In “Experiment 2”, the impact of emotion induced by the chords was correlated with rhythmic factors, specifically tempo and syncopation. As music in the real world frequently comprises multifaceted factors, such as melody, chords, and rhythm, this attempt can elucidate groove induction in everyday listening and provide an additional aspect of body responses toward music that can induce specific emotions.

## Experiment 1

### Methods

#### Participants

Seventy participants (37 women, 33 men) aged 18–60 years (median = 20 years) participated in the online study. Fifty-six participants had prior musical experience. The most commonly used musical instrument was the piano, which was played by 40 participants. Seven participants were current or former music majors. I recruited participants through acquaintances and their connections. Two participants were excluded from the participant group because their ratings incorporated the same numbers for the nine and seven stimuli.

#### Stimuli

Nine stimuli created by a professional composer were used. These stimuli comprised four types of piano chord progressions (C1–C4), a rhythmic pattern without chord progressions (i.e., drum break) only (R), and four types of piano chord progressions with the same drum breaks (RC1–RC4). The composer was asked to create a chord progression for the four stimuli that gradually changed from happy (C1) to sad (C4). C1–C4 were composed of E major, D major, A minor, and C minor, respectively. This study operationally defined happiness/sadness as valence, although valence does not directly refer to happiness/sadness (Binyamin-Suissa et al., 2021). I asked a professional composer to arrange the timbre and loudness of each instrument naturally. Subsequently, I and the composer first confirmed whether the stimuli evoked the intended emotion before the experiment.

**Fig. 1:**
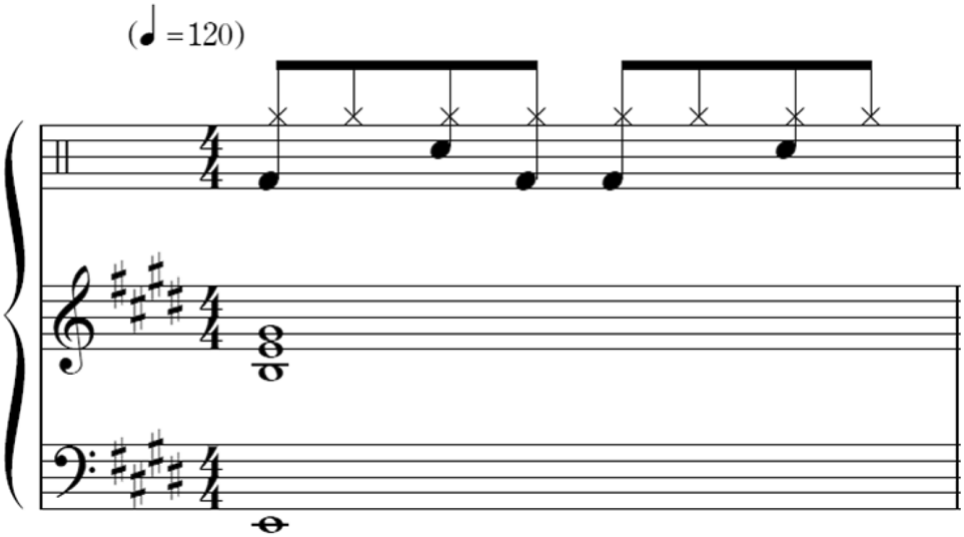
An example of the first measure of RC1. The score appearing on the uppermost represents drum breaks. The top, middle, and bottom rows represent the hi-hat, snare drum, and bass drum, respectively

To confirm emotions that were supposed to be induced by chord progressions only, four out of nine stimuli comprised repeating eight-chord progressions played only on the piano, that is, without drum breaks. A chord occurred per 2.4 s, which meant that the piano sound occurred at the first beat of four quarters, at 100 BPM. One stimulus comprised a rhythm pattern played by drums only and a typical rock drum break with a tempo of 120 BPM. The four stimuli comprised the aforementioned four-chord progressions with the aforementioned drum breaks at a tempo of 120 BPM (Fig. 1). The entire RC1 score is shown in Appendix 1. To investigate the influence of chords on groove, I aligned the tempo for comparison between drum breaks with and without chords, that is, RC1–RC4 and R. In these stimuli, the chord played by the piano sounds only at the first beat of the fourth quarter. Each stimulus was 51 s long and included 1 s of fade-out.

#### Procedure

The experiment was conducted using an online survey (https://www.soscisurvey.de). Participants provided information about their attributes, namely, age, sex, and musical experience. Subsequently, they practiced in a trial session and performed the actual session. Four combinations of randomized stimuli were used. Participants randomly rated one of them. In the description of the instructions, I instructed the participants that they were allowed to rate items before listening to the end of the stimuli because each stimulus consisted of a repeated meter. Participants were asked to adjust the volume in line with their appropriate loudness. Participants were free to use listening devices, such as headphones and speakers, because of the ecological validity of listening to music through various types of devices in daily life.

Participants responded to nine items for each stimulus using a seven-point scale (from 1 = do not agree at all to 7 = strongly agree). The questionnaires comprised items based on previous studies on groove (Etani et al., 2018; Janata et al., 2012; Labbé and Grandjean 2014; Madison, 2006), items regarding induced emotions, and liking. Thus, participants were instructed to rate how they felt about each given stimulus concerning the following nine items: urge to move, feeling *nori*, feeling pleasure, resonating with the rhythm, feeling happy, feeling sad, feeling tender, feeling fear, and liking each stimulus. In the present study, the results of the item, “urge to move” were considered as groove ratings (Etani et al., 2018; Janata et al., 2012; Madison, 2006). The entire experiment lasted approximately 10–15 min.

#### Ethical statement

All participants were informed that their participation was voluntary, that they were free to quit the survey, and that the data would be anonymized. The first informed consent page was displayed on the website of the online survey. This study was approved by the ethics committee of the Graduate School of Osaka University, Japan.

### Results

**Fig. 2:**
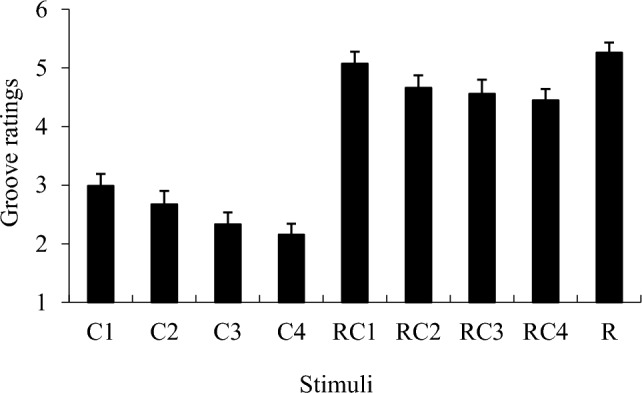
Mean groove ratings. “C” represents chord progression only, “RC” represents chord progression with the rhythmic pattern (drum breaks), and “R” represents the rhythmic pattern (drum breaks) only. For example, RC1 means C1 chord progression with drum breaks. Error bars represent standard errors

The average groove ratings are shown in Fig. 2. The average ratings of all items are shown in Table S1. To investigate the effects of chord progressions and the presence of drum breaks, a two-way repeated-measures ANOVA (4 chords × with/without drum breaks) was conducted. The main effect of chords progressions (*F*(2.37, 168.83) = 9.113, *p* < 0.001, *η*_p_^2^ = 0.117; Greenhouse–Geisser correction under the assumption of the violation of sphericity), and the main effect of the presence of drum breaks (*F*(1, 69) = 87.458, *p* < 0.001, *η*_p_^2^ = 0.556) were significant. However, this interaction was not significant (*p* = 0.73).

Multiple comparisons revealed that groove ratings for stimuli with C1 were rated significantly higher on groove than the other three (*p*s < 0.05). Stimuli with C2 were also higher than those with C4 (*p*s < 0.05). The stimuli with drum breaks were higher than those without drum breaks.

To compare the groove ratings of the drum breaks only (i.e., R in Fig. 2), a *t*-test (R versus others) was conducted. The ratings of R were significantly higher than that of C1–C4 and RC2–RC4 (*p*s < 0.01).

**Fig. 3:**
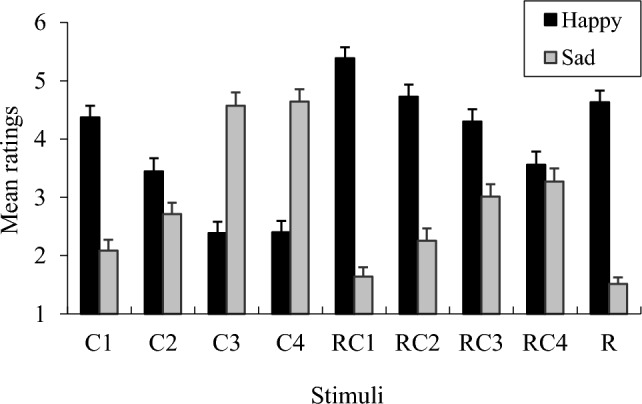
Mean happiness and sadness ratings. Error bars represent standard errors

To confirm whether chord progressions altered the induced emotions, I examined the mean ratings of emotions. The mean happiness and sadness ratings for each stimulus are shown in Fig. 3. From C1–C4, happiness ratings decreased, whereas sadness ratings increased. Consequently, the chord progressions used in the present study induced the emotions of the participants as intended. Remarkably, regarding drum patterns alone, the happiness ratings were the same as those of the happiest stimuli, whereas the sadness ratings in drum patterns were low.

**Fig. 4:**
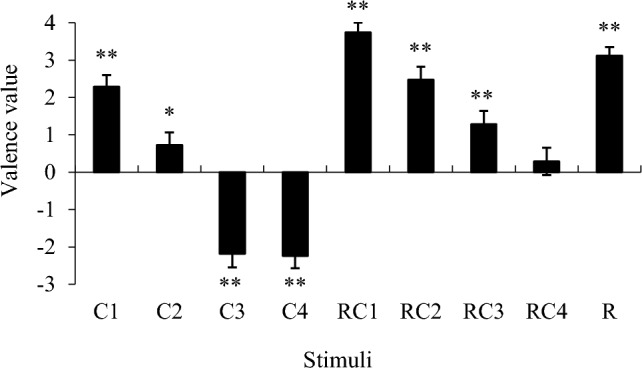
Mean value of valence indices. Error bars represent standard errors. **p < 0.01, *p < 0.05

To simplify the results, as an indicator, I defined the numerical difference as the valence value by subtracting the sadness ratings from the happiness ratings (Fig. 4). A one-sample *t*-test compared with zero was conducted. The results showed that C1 and C2 were significantly positive, whereas C3 and C4 were significantly negative. Hence, the chord progressions in this experiment were able to evoke the intended emotions. In addition, as an overall tendency, the valence ratings tended to be biased toward positive emotions when the stimuli comprised drum breaks.

**Fig. 5:**
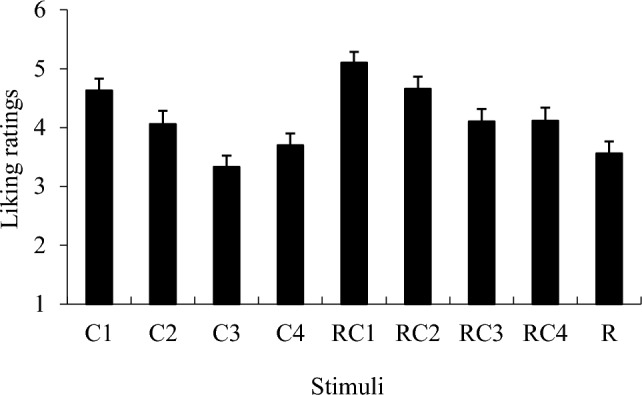
Mean ratings of Liking. Error bars represent standard errors

Figure 5 shows the ratings of the results for liking the stimuli. To compare the liking ratings on accompanying chord progressions, a *t*-test between the stimulus which contained only drum breaks (i.e., “R”) and other stimuli was conducted. The results showed that the rating of stimulus “R” was significantly lower than that of C1–C2 and RC1–RC4 (*p*s < 0.05). This indicates that the ratings of liking were not considerably high for drum patterns, although the groove ratings were high.

To examine the relationship between groove and other rating items, I calculated correlation coefficients using the average ratings of each stimulus (Table 1). The results showed significant positive correlations between groove ratings and happiness ratings but negative correlations between groove ratings and sadness and fear ratings. Furthermore, groove ratings showed a significant positive correlation with the rating of *nori*, which is frequently described as groove in Japan and is related to the sensation of vertical and horizontal movement while listening to music (Etani et al., 2018; Kawase & Eguchi, 2010) and resonating with rhythm, but a mild positive correlation with pleasurable ratings.

**Table 1:** Correlation coefficients between variables

	1	2	3	4	5	6	7	8
1. Groove								
2. Nori	1.00**							
3. Pleasurable	0.59^†^	0.57						
4. Resonating	0.74*	0.73*	0.93**					
5. Happy	0.83*	0.82*	0.93**	0.92**				
6. Sad	− 0.73*	− 0.69*	− 0.89**	− 0.81*	− 0.93**			
7. Tender	0.06	0.05	0.79*	0.64^†^	0.54	− 0.52		
8. Fear	− 0.71*	− 0.68*	− 0.90**	− 0.80*	− 0.93**	0.99**	− 0.57	
9. Liking	0.42	0.43	0.89**	0.86**	0.75*	− 0.62^†^	0.89**	− 0.67^†^

*p < 0.05

**p < 0.01

^†^*p* < 0.1

### Discussion

The results showed that the higher the level of happiness represented by the chord progressions became, the higher the groove ratings emerged, even when the drum breaks were the same. In addition, the groove ratings for stimuli with drum breaks were higher than those for stimuli created only by chord progressions without drum breaks. These results highlight two significant points. First, happy/sad chord progressions strongly affected groove ratings, regardless of rhythmic patterns. Second, rhythmic patterns are essential in facilitating groove ratings.

Nevertheless, despite these findings, it remains unclear whether the chord progressions interact with other rhythmic factors. Given that various musical factors, such as tempo, influence emotional characteristics (Juslin & Timmers, 2010), interactions between emotions induced by chord progressions and rhythmic factors may affect groove ratings. For example, it seems reasonable that a slow piece is groovier than a fast piece when it induces a sad emotion, as a slow tempo is usually employed for sad pieces (Hunter et al., 2010; Juslin & Timmers, 2010). In other words, depending on the emotional characteristics of a piece, the optimal value of rhythmic features that induce groove may differ. Thus, Experiment 2 was conducted to test the associations between emotions derived from chord progressions, tempo, and syncopation that influenced groove ratings (Etani et al., 2018; Witek et al., 2014).

## Experiment 2

To examine how groove ratings are influenced by the associations between emotions induced by chord progressions and rhythmic pattern factors, an online survey was conducted using combinations of (1) two types of chord progressions (happy and sad), (2) two types of tempi (medium and slow), and (3) two types of syncopation levels (medium and low).

### Methods

#### Participants

Seventy participants (43 women, 27 men) aged 18–59 years (median = 21 years) participated in the online study. A total of 48 participants had prior musical experience. The most commonly used musical instrument was the piano, which was played by 30 participants. Sixteen participants answered that they are majoring or majored in music. I recruited participants from my acquaintances.

#### Stimuli

**Fig. 6:**
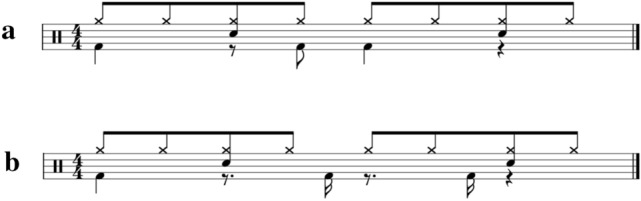
Drum breaks employed in Experiment 2. **a** and **b** represent low and medium syncopated drum breaks. These were employed by Etani et al. (2018)

Experiment 2 uses 12 stimuli created by a professional composer, involving eight stimuli consisting of drum breaks and chord progressions by the piano sound, that is, two emotions (happy and sad chord progressions; C1 and C4 in experiment 1) × two tempi (medium and slow tempo; 120 BPM and 80 BPM) × two rhythmic patterns (low and medium levels of syncopation; Fig. 6). In addition, four rhythmic patterns were without chord progressions created, including combinations of two tempi (120 and 80 BPM) and two rhythmic patterns (low and medium levels of syncopation). The volume balance of the instruments was the same as that used in Experiment 1. As in Experiment 1, each stimulus was 51 s long, including 1 s of fading.

#### Procedure

The process of Experiment 2 was the same as that of Experiment 1 except for differences in the types of stimuli. In other words, the experiment was conducted as an online survey, employing the same instructions and items. This study was approved by the ethics committees of the Graduate School of Osaka University and Kobe Gakuin University.

### Results

**Fig. 7:**
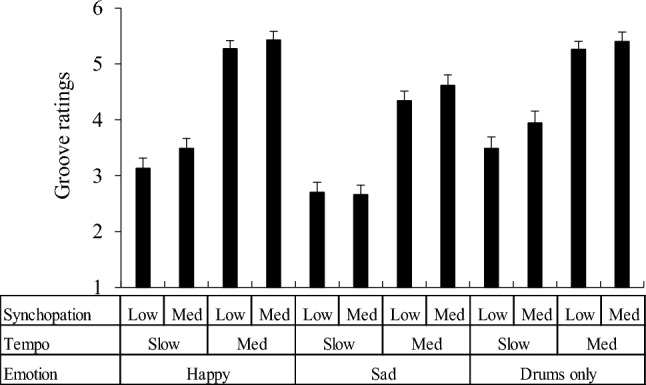
Mean groove ratings. Error bars represent standard errors

The average groove ratings for each stimulus are shown in Fig. 7. The average ratings of all items are shown in Table S2. A three-way ANOVA (emotion × tempo × syncopation) was conducted. Main effect of emotion (*F*(2,138) = 27.973, *p* < 0.001, *η*_p_^2^ = 0.288), tempo (*F*(1,69) = 201.069, *p* < 0.001* η*_p_^2^ = 0.745), and syncopation (*F*(1, 69) = 8.081, *p* = 0.006, *η*_p_^2^ = 0.105) were significant. Stimuli with sad chords showed significantly lower groove than those with happy chords or without chords (*p* < 0.001). Medium-tempo stimuli showed high groove (*p* < 0.001), and more syncopated stimuli also showed high groove. Interaction between emotion and tempo showed a significant tendency (*F*(2,138) = 2.576, *p* = 0.080, *η*_p_^2^ = 0.036). Other interactions among emotional characteristics, tempo, and syncopation were not observed. In the slow tempo condition, the stimuli without chord progressions were the grooviest, and those with sad chords were the least groovy (*p* < 0.05). In the medium-tempo condition, stimuli with a sad chord were also the worst groovy (*p* < 0.001). However, the stimuli with happy chords and those without chords showed the same groove level. Under all emotional conditions, all stimuli with a medium tempo were groovier than those with slow tempi.

The correlation coefficients were calculated using the mean value of each item (Table 2). As in Experiment 1, groove showed a significant positive correlation with happiness and a significant negative correlation with sadness. Groove was also significantly correlated with *nori*.

**Table 2:** Correlation coefficient between variables

	1	2	3	4	5	6	7	8
1. Groove								
2. Nori	0.99**							
3. Pleasurable	0.41	0.46						
4. Resonating	0.89**	0.92**	0.73**					
5. Happy	0.82**	0.82**	0.81**	0.89**				
6. Sad	− 0.61*	− 0.53^†^	− 0.45	− 0.48	− 0.76**			
7. Tender	− 0.07	− 0.01	0.85**	0.35	0.43	− 0.14		
8. Fear	− 0.59*	− 0.52^†^	− 0.57^†^	− 0.52^†^	− 0.79**	0.98**	− 0.27	
9. Liking	0.48	0.55^†^	0.88**	0.81**	0.72**	− 0.17	0.75**	− 0.27

*p < 0.05

**p < 0.01

^†^*p* < 0.1

### Discussion

The present study showed that chord progression, tempo, and syncopation had significant impacts on groove, although marginal interactions between chord progress and tempo were observed. Given these results, the influence of emotion induced by chord progressions on groove rarely interacts with rhythmic characteristics, specifically tempo and syncopation. In other words, a slow tempo with a sad musical piece did not make listeners’ perceptions groovier than a fast tempo with a sad musical piece.

## General discussion

This study aimed to examine whether emotions expressed by musical chord progressions change groove. For the online listening survey in Experiment 1, I used stimuli consisting of chord progressions created for expressions of different valence levels and stimuli consisting of chord progressions with drum breaks. To examine the interactions between emotional aspects and rhythmic characteristics when groove emerges, Experiment 2 investigated the associations among chord progression, tempi, levels of syncopation, and groove ratings. The primary findings were as follows: (1) chord progressions designed to evoke happiness were more likely to induce groove; (2) emotional characteristics did not interact with tempi and syncopation in terms of groove ratings; and (3) the accompaniment of drum breaks enhanced groove in both happy and sad chord progressions. These results suggest that musical pieces whose chord progressions induced happiness were more likely to evoke higher levels of groove. These findings also provided clues for the comprehension of two unclear associations between: (a) emotions evoked by chord progression (Gagnon & Peretz, 2003; Labbé et al., 2021) and groove inductions accompanying pleasurable feelings (Etani et al., 2023; Hosken, 2020; Janata et al., 2012; Stupacher et al., 2016; Witek et al., 2014), and (b) emotional factors provided by non-rhythmic features (Lahdelma & Eerola, 2016) and groove related to rhythmic features (Etani et al., 2018; Witek et al., 2014).

### Happy chord progressions facilitate groove inductions

Chord progressions designed to evoke happiness promoted more groove than those designed to evoke sadness in Experiments 1 and 2. Consequently, elements within musical pieces that may induce emotions can influence groove. Groove induction is accompanied by positive emotions such as pleasure and enjoyment (Janata et al., 2012; Kawase & Eguchi, 2010; Witek et al., 2014). This suggests that the rhythm itself evokes positive emotions. By contrast, in this study, musical factors that induced emotions changed groove levels, even when the rhythmic patterns were the same. In particular, changes in the valence of chord progressions altered groove without changes in rhythmic factors, whereas changes in drum breaks altered groove and happiness ratings. This indicates that not only rhythms that provoke the urge to move induce enjoyment but also the elements that induce enjoyment provoke the urge to move our body.

Prior studies have shown that the emotional characteristics of pieces modulate body movements (Burger et al., 2012; Janata et al., 2012). Controlling each level of happiness of musical pieces independently changes groove, as commercially available musical pieces provide a correlation between groove and enjoyment (Janata et al., 2012), and the emotional characteristics of musical pieces shift body movement (Burger et al., 2012). Burger et al. (2012) suggested that songs that evoke happiness are related to the rotations of the body and highly complex body movements in three dimensions. Considering these findings, it can be expected that groove changes with variations in the emotional characteristics of musical pieces. However, most studies use commercially available songs that typically fluctuate in relation to various factors. For example, a sad song will have chords, tempi, and rhythms that differ from those of happy songs.

Meanwhile, the present results showed that chord progressions that induced happiness provided higher levels of groove. This result shows a new aspect of the association between groove and emotion because it was unclear whether urge to move was affected by factors that evoke pleasant feelings, although previous research has shown that the urge to move and pleasant feelings co-occur (Etani et al., 2018; Hosken, 2020; Janata et al., 2012; Stupacher et al., 2016; Witek et al., 2014). Thus, the present results provide a new perspective on the association between emotional processing and embodiment.

### Possible explanations for emotional chord progressions affecting groove

Why were groove ratings changed by happy/sad chord progressions with the same drum breaks? One possible explanation for this result is the relationship between the valence produced by chord progressions and arousal. The happiness of musical pieces can induce higher levels of arousal (Salimpoor et al., 2009). The major mode, which is related to happy music (Gagnon & Peretz, 2003), induced higher levels of arousal than the minor chord (Labbé et al., 2021). Thus, music designed to induce happiness in the present study may have evoked higher levels of arousal than sad music. Bowling et al. (2019) suggested that high-groove music generated higher levels of arousal. As several types of chord progressions in the present study with controlled valence were able to induce emotions as designed, the stimuli used in the present study could also induce arousal. Accordingly, happy music induces high arousal, which may in turn induce groove related to arousal.

Another possible explanation is the flow state, which is related to happiness (Csikszentmihalyi, 2013). As groove can be related to flow states (Janata et al., 2012), music designed to induce happy feelings may connect happy feelings, flow, and groove in a circle. Otherwise, the brain’s reward system may be a key link between happy music and groove. Experienced groove is related to the brain’s reward system and motor networks (Matthews et al., 2020), while enjoyable music is associated with the reward system (e.g., Koelsch et al., 2006). This suggests that happiness evoked by chord progressions may be linked to groove induction through the reward system. Thus, happy music can be a useful cue for the scrutiny of correlations between groove and proximate status, namely, arousal, flow, and activation of the reward system.

### Interactions among emotional chord progression, tempo and syncopation for groove

Emotional characteristics (happiness and sadness) rarely interacted with syncopation or tempo in terms of groove ratings. Accordingly, tempo, syncopation, and chords independently affect groove, although several acoustic characteristics can interact with each other (Matthews et al., 2019; Witek et al., 2014). In Etani et al.’s (2018) study of groove, the higher the event density, the slower the optimal tempi is. This finding suggests that to maintain the optimal event density, the optimal tempi for groove induction in 16-beat patterns became slower because the event density in 16-beat patterns was essentially high. This suggests that groove may involve the interaction of multiple musical elements.

By contrast, for groove ratings in the present study, the valence of chord progressions was rarely associated with tempo and syncopation. Considering that the tempo of sad music can typically be slow (Hunter et al., 2010; Juslin & Laukka, 2004; Juslin & Timmers, 2010), it may be reasonable that the optimal tempo for sad groovy music was slower than that for happy music. However, the results of the present study contradict this hypothesis. In other words, a slower tempo did not become groovier even if chord progressions designed to evoke sad emotions were played. One possible reason for this result is that the musical factors of rhythm and chord progressions did not interact with each other for groove induction because chord progressions did not influence rhythmic aspects. Consequently, musical pieces designed to evoke happy emotions were rated as groovier regardless of syncopation and tempo. This suggests that chords affect groove in a different way than rhythm.

This study also supports Senn et al.’s model of groove, in which musical properties cause pleasure, which mediates the urge to move (Senn et al., 2019). It also shows that groove can be changed by manipulating pleasure using musical elements unrelated to rhythm. This result suggests that although, as shown in previous studies, pleasure accompanies groove induced by rhythm, groove is also evoked by the occurrence of pleasure regardless of rhythm. This implies that the sensation of the urge to move the body causes pleasure and vice versa; that is, pleasure also causes the sensation of wanting to move the body.

### Impacts of rhythmic features on groove

Drum breaks promote groove in both happy and sad chord progressions. Combinations of happy chord progressions and drum breaks were rated as groovy enough to the extent that they did not interfere with the groove induced by drum breaks alone. In other words, the effects of rhythm on groove could be stronger than emotional chord progressions, although combinations of the same rhythmic patterns and different emotional chord progressions changed groove levels. Meanwhile, when sad chord progressions are added, which can interfere with the positive valence, the groove levels may decrease. Thus, adding a taste of happiness to drum breaks does not greatly promote groove; rather, it seems appropriate to say that sad elements suppress groove. This suggests that the impact of rhythm on groove is significant as groove ratings were similar in both chord progressions designed to induce happy emotions and drum breaks alone in both Experiments 1 and 2. In Experiment 1, drum breaks alone had a significantly positive valence, and the addition of drum breaks evoked more positive emotions. This result is in line with findings on the impact of rhythmic patterns on groove (Etani et al., 2018; Frühauf et al., 2013; Sioros et al., 2014; Witek et al., 2014).

Meanwhile, the liking ratings were lower for drum breaks alone than for those with chord progressions. This indicates that higher levels of groove ratings do not directly lead to a preference for musical pieces. These results are inconsistent with previous findings on the relationship between groove and musical preference (Kowalewski et al., 2020; Senn et al., 2021). One possible explanation for this is that the present study highlighted the emotional characteristics of musical pieces and groove indications, whereas prior studies investigated the familiarity or preference for musical pieces and groove.

### Limitations

A limitation of this study is that only a few musical factors that can influence groove, such as chords, tempo, and syncopation, were examined. As real-world music consists of many complex acoustical characteristics, it may be useful to investigate how tempo, syncopation, and emotions induced by chords affect groove and how they relate to the listener’s preferences.

## Conclusion

These findings provide new evidence supporting the impact of the emotional characteristics induced by chord progressions on groove. In addition, by controlling for each musical factor, this study revealed the extent to which a single factor affected groove and there is no interaction between them. Furthermore, the results of this study suggest a bidirectional relationship between the sensation of the urge to move and the emotional characteristics evoked by music. The results can apply to real-world listening situations where complex elements are included in music pieces, for example, effective groovy music for rehabilitation (Leow et al., 2021), dance (Vander Elst et al., 2021), and composing songs to improve execution functions (Fukuie et al., 2022).

In future studies, further speculations on the relationships between emotional expressions and groove may provide new aspects of embodiment in music as well as physical activities, such as dance or exercise. This can provide a cue to determine the reasons why dance music is not solely composed of rhythm, from ancient ritual music to contemporary music. A cross-cultural study of groove (Etani et al., 2018; Kawase & Eguchi, 2010; Witek et al., 2020) is also beneficial for investigating whether emotions and groove indications are universal.

### Supplementary Information

Below is the link to the electronic supplementary material.

Supplementary file1 (JPG 879 KB)

Supplementary file2 (PDF 93 KB)

## Data Availability

All data and materials can be available from the corresponding author on reasonable request.
