# Supplementary figures and images for: Is happier music groovier? The influence of emotional characteristics of musical chord progressions on groove
Source: Psychol Res. 2023 Aug 24;88(2):438–48. doi: 10.1007/s00426-023-01869-x (PMC10858120; doi:10.1007/s00426-023-01869-x)

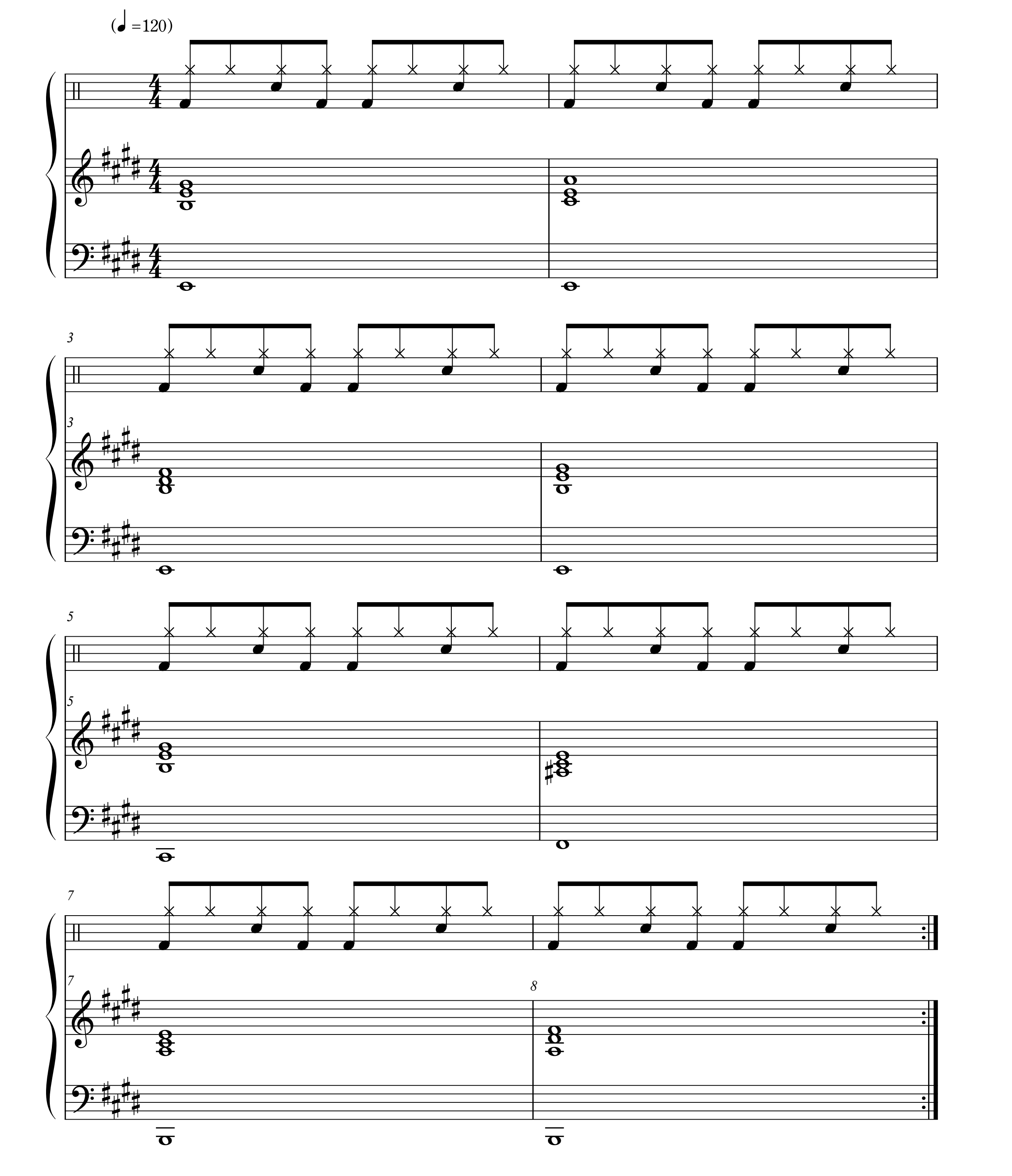

Supplement: Supplementary file 1 — Supplementary file1 (JPG 879 KB) [file 426_2023_1869_MOESM1_ESM.jpg]
