# Supplementary material for: Is happier music groovier? The influence of emotional characteristics of musical chord progressions on groove
Source: Psychol Res. 2023 Aug 24;88(2):438–48. doi: 10.1007/s00426-023-01869-x (PMC10858120; doi:10.1007/s00426-023-01869-x)
Supplement: Supplementary file 2 — Supplementary file2 (PDF 93 KB) [file 426_2023_1869_MOESM2_ESM.pdf]

Table S1. Mean ratings of each item in Experiment 1.

	C1	C2	C3	C4	RC1	RC2	RC3	RC4	R
Groove	3.0 (1.7)	2.7 (1.9)	2.3 (1.7)	2.2 (1.5)	5.1 (1.7)	4.7 (1.8)	4.6 (2)	4.4 (1.7)	5.3 (1.5)
Nori	2.9 (1.6)	2.6 (1.8)	2.3 (1.7)	2.2 (1.6)	5.2 (1.6)	4.8 (1.6)	4.9 (1.7)	4.6 (1.6)	5.2 (1.7)
Pleasurable	5.1 (1.6)	4.3 (1.8)	3.6 (1.6)	3.7 (1.7)	5.4 (1.6)	4.8 (1.6)	4.3 (1.6)	4.1 (1.8)	4.5 (1.7)
Resonating	4.5 (1.6)	3.6 (1.7)	3.4 (1.6)	3.4 (1.7)	4.9 (1.6)	4.5 (1.6)	4.1 (1.7)	4.2 (1.6)	4.1 (1.8)
Happy	4.4 (1.7)	3.4 (1.9)	2.4 (1.6)	2.4 (1.6)	5.4 (1.6)	4.7 (1.7)	4.3 (1.8)	3.6 (1.9)	4.6 (1.7)
Sad	2.1 (1.6)	2.7 (1.6)	4.6 (1.9)	4.6 (1.8)	1.6 (1.3)	2.3 (1.8)	3.0 (1.8)	3.3 (1.9)	1.5 (0.9)
Tender	4.7 (1.9)	4.1 (1.9)	3 (1.6)	3.1 (1.5)	4.6 (1.8)	4.2 (1.7)	3.4 (1.7)	3.4 (1.5)	2.8 (1.8)
Fear	1.7 (1.2)	1.8 (1.3)	3.1 (2)	3.0 (2)	1.4 (1.1)	1.7 (1.3)	2 (1.4)	2.3 (1.7)	1.5 (0.9)
Liking	4.6 (1.6)	4.1 (1.7)	3.3 (1.8)	3.7 (1.8)	5.1 (1.7)	4.7 (1.5)	4.1 (1.7)	4.1 (1.9)	3.6 (1.5)

Values in parentheses represent standard deviations.

Table S2. Mean ratings of each item in Experiment 2.

Emotion	Happy				Sad				Drums only			
Tempo	Slow		Mid		Slow		Mid		Slow		Mid	
Synchopation	Low	Mid	Low	Mid	Low	Mid	Low	Mid	Low	Mid	Low	Mid
Groove	3.1 (1.5)	3.5 (1.5)	5.3 (1.2)	5.4 (1.3)	2.7 (1.5)	2.7 (1.4)	4.3 (1.4)	4.6 (1.6)	3.5 (1.8)	3.9 (1.8)	5.3 (1.2)	5.4 (1.4)
Nori	3.2 (1.5)	3.6 (1.4)	5.5 (1.1)	5.5 (1.3)	2.7 (1.5)	2.7 (1.4)	4.7 (1.3)	4.8 (1.4)	3.1 (1.6)	3.8 (1.7)	5.4 (1.3)	5.5 (1.3)
Pleasurable	5.0 (1.5)	5.1 (1.4)	5.3 (1.3)	5.1 (1.4)	3.7 (1.6)	3.6 (1.6)	4.0 (1.5)	4.1 (1.6)	3.4 (1.6)	3.7 (1.7)	4.3 (1.4)	4.1 (1.7)
Resonating	3.7 (1.4)	3.9 (1.5)	4.6 (1.4)	4.6 (1.4)	3.1 (1.4)	3.2 (1.4)	3.9 (1.6)	4.3 (1.6)	3.2 (1.5)	3.4 (1.7)	4.1 (1.6)	4.2 (1.7)
Happy	3.8 (1.5)	4.1 (1.5)	5.4 (1.2)	5.5 (1.3)	2.2 (1.3)	2.1 (1.1)	3.1 (1.8)	3.3 (1.7)	2.7 (1.6)	3.3 (1.7)	4.4 (1.6)	4.7 (1.6)
Sad	2.5 (1.6)	2.1 (1.4)	1.5 (0.9)	1.6 (0.9)	4.6 (1.8)	4.8 (1.7)	3.9 (1.8)	3.8 (1.7)	1.8 (1.1)	1.7 (1)	1.6 (1)	1.4 (0.9)

Tender	5.0<br>(1.6)	4.8<br>(1.6)	4.5<br>(1.5)	4.4<br>(1.6)	3.0<br>(1.5)	2.9<br>(1.5)	3.0<br>(1.5)	3.0<br>(1.6)	2.5<br>(1.5)	2.4<br>(1.5)	2.3<br>(1.3)	2.3<br>(1.4)
Fear	1.5<br>(1.0)	1.5<br>(1.1)	1.3<br>(0.7)	1.4<br>(0.8)	3.1<br>(1.7)	3.3<br>(1.8)	2.5<br>(1.6)	2.6<br>(1.7)	1.6<br>(1)	1.6<br>(1.1)	1.4<br>(0.9)	1.4<br>(1)
Liking	4.3<br>(1.6)	4.3<br>(1.5)	5.0<br>(1.4)	5.0<br>(1.3)	3.7<br>(1.6)	3.3<br>(1.7)	3.8<br>(1.6)	4.4<br>(1.7)	2.9<br>(1.5)	2.9<br>(1.7)	3.7<br>(1.5)	3.9<br>(1.6)

Values in parentheses represent standard deviations.
